# Study on the dynamic characteristics of rock surrounding a wellbore in energy storage areas during deep geothermal energy mining

**DOI:** 10.1371/journal.pone.0237823

**Published:** 2020-08-21

**Authors:** Chun Wang, Huai-bin Wang, Mei-zhi Xie, Zu-qiang Xiong, Cheng Wang, Lu-ping Cheng, Shuai-fei Zhan

**Affiliations:** 1 School of Energy Science and Engineering, Henan Polytechnic University, Jiaozuo, Henan, China; 2 Guangxi Key Laboratory of Disaster Prevention and Engineering Safety, Guangxi University, Nanning, Guangxi, China; 3 State and Local Joint Engineering Laboratory for Gas Drainage & Ground Control of Deep Mines, Henan Polytechnic University, Jiaozuo, Henan, China; 4 The Collaborative Innovation Center of Coal Safety Production of Henan, Jiaozuo, Henan, China; 5 College of Civil Engineering and Architecture, Guangxi University, Nanning, Guangxi, China; China University of Mining and Technology, CHINA

## Abstract

Based on the engineering background in which the rock surrounding a wellbore is affected by a thermal shock, impact disturbances from drilling vibration, cyclic heat extraction and high temperature during hydrothermal geothermal energy mining, the environmental conditions in the shaft wall rock are simulated by means of high temperature, cooling, immersing granite in water with different curing temperatures and applying impact loads. Additionally, an experimental study on the mechanical characteristics of circular granite specimens under radial impact loads and in the heat treatment and water curing conditions is carried out. The results show that the inner diameters of the rings, heating temperatures, curing water temperatures and cycle heating times are less affected than other parameters by the impact load-strain curves of circular granite, which can generally be divided into three sections, i.e., the initial straight stage, nonlinear ascent yield stage and post-peak nonlinear decline stage. The factors in the test weaken the capacity of the circular granite to resist the impact, but the sizes of the inner diameters of the rings play a leading role. Dynamic tensile strain is generated in the inner wall along the impact direction during the impact, while compressive strain is produced on the inner wall in the vertical impact loading direction. By analysing the crack propagation and final failure mode of circular granite, it is found that dynamic tensile failures are generated, crack initiation starts from the inner wall along the impact loading direction, and the outer circle in the vertical direction lags behind. The crack starts early and develops quickly on one side of the transmission bar. Finally, the failure criterion is established on the basis of some assumptions and circular-granite deformation failure characteristics, and the parameters, measured by the Brazilian disk test, are reasonably verified via substitution into the failure criterion equation.

## 1. Introduction

Rock is a special geological material that has the properties of non-uniformity, discontinuity and anisotropy. The exploitation of resources for living, such as coal, oil, and natural gas, depends on the drilling, excavation, and support of rocks [1,2]. With the increase in human energy consumption, traditional resources are gradually unable to meet the development of human society, and the exploitation of clean and renewable resources [3,4] is an urgent need. Deep geothermal energy is considered a new energy source that is a clean, renewable and potential resourcefor solving the energy crisis. However, the critical problem is how to extract deep geothermal energy safely, efficiently and sustainably [5,6]. Currently, the methods to explore geothermal energy include single wellbore heat transforming, CO_2_ geological storage, double-well circulation exploration and so on [7–9], and they all require drilling deep wells. Therefore, the efficiency of deep geothermal energy extraction can be effectively improved by obtaining the mechanical properties of rocks under conditions of high temperature and impact.

Researchers have performed many studies on the unique mechanical conditions that rocks experience during the production of geothermal energy. It was found that the dynamic tensile failure strain of rocks increased with increasing temperature [10,11], and the dynamic tensile strength first increased and then decreased by using Brazilian discs to perform dynamic splitting tests of high-temperature rocks [12,13]. Some researchers also carried out studies on the rock surrounding wellbores under a high static load, and it was found that the dynamic tensile strength of the rock under a high static load was far greater than the tensile strength under a static load, and the failure characteristics of tensile strength mentioned above were generally consistent [14,15]. When the rocks have been subjected to the coupling effects of temperature and pressure, the dynamic tensile strength first increased and then decreased with increasing temperature and static load, and the failure process was rather aggressive [16,17]. When rock specimens were in a high-temperature circular process, internal micro-cracks noticeably developed with increasing thermal cycle time, but there was no obvious directionality, indicating that the high-temperature cycle destroyed the original structure inside the rock and caused thermal damage to the rock [18–20]. Considering that the rock surrounding wellbores was affected by drilling vibration, thermal impact and earthquake waves and that tensile failure frequently occurred in the wellbore, dynamic Brazilian disc splitting tests were carried out. The cumulative damage of the rock increased exponentially with increasing strain rate, the ultimate failure modes of the disc rock samples were closely related to the impact velocity, and the dynamic tensile strengths measured by the test were far larger than the static tensile strengths [21–24]. Analysing the above studies, it was found that high temperature, high in situ stress and dynamic disturbances were taken into account, but the influence of the cross section of high-depth wellbores, medium water and cycle heat recovery times was overlooked.

To investigate the failure mechanism of the surrounding rock in a high-depth wellbore, the mechanical characteristics of circular rock samples under a radial impact load were carried out under the assumption that there was a set of maximum horizontal forces in a certain horizontal direction and that it was the direct factor leading to the failure of the rock surrounding the wellbore. Some researchers have investigated the mechanical characteristics of circular specimens and found that the maximum radical load that circular specimens could bear decreased with increasing inner diameter and increased with increasing inner circle eccentricity and that the water condition could affect the ability of circular specimens to resist the radial compression load, but the influence of thermal impact and drilling vibration shock was overlooked [25–27]. Based on the above studies and the environment that mining wells and recharge wells experience during the exploitation of geothermal energy, a mechanical test study of circular granite under a radial impact load and in the heat treatment and water curing conditions was carried out to predict the dynamic failure of circular granite specimens, which would provide a theoretical reference for seeking methods to extend the service life of geothermal wells.

## 2 Experimental preparation for the heat treatment and water curing compression test

### 2.1 Specimen preparation

The granite used in the test was taken from a granite body at a depth of approximately 500 m in the Biyang granite mine. The Biyang granite mine is located in Biyang County, Henan Province, China. Scholars are welcome to conduct academic research in mines. In this paper, only rock samples were taken from the mine, and there was no conflict of interest. Test sampling was approved by the Biyang granite mine; therefore, no special permission was required for this research activity. To conduct an experimental study on the mechanical characteristics of the radial impact of circular granite in the heat treatment and water curing conditions, the specimens were drilled from granite blocks with good integrity and homogeneity. The cylindrical specimens were prepared with a diameter of 50 mm and a length of 30 mm. The circular granite specimens were prepared with inner diameters of 6 mm, 12 mm, 18 mm, and 22 mm; outer diameters of 50 mm; and a thickness of 30 mm. To ensure that the strains on both ends of the granite specimens under the radical impact load were precisely measured, both ends of the rock specimens were carefully treated to ensure that their non-parallelism and non-verticality were no more than 0.02 mm.

### 2.2 Experimental apparatus

**Fig 1:**
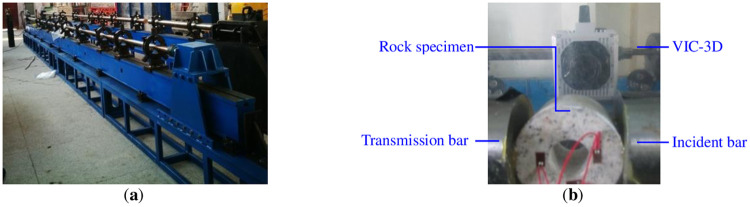
Mechanical test system with radial impact of circular granite specimens. (**a**) SHPB and (**b**) VIC-3D.

The test system consisted of a SHPB impact mechanics test system and a VIC-3D non-contact full-field strain measurement system. Pictures of the devices are shown in Fig 1.

A split Hopkinson pressure bar system was used to test the radical impact load. The lengths of the incident bar and transmitted bar were3 m, and the length of the bullet was0.4 m. VIC-3D, which consisted of a high-speed camera, lighting system, trigger, synchronization control system and computational analysis system, was used to detect the strain on one end of the circular granite specimens in the process of compression. The experimental system was also equipped with a DH8302 high-performance dynamic strain gauge and was used to detect the strain of the inner ring wall along the radical impact load direction and over the radical impact load direction on another end of the circular granite specimens during the test.

### 2.3 Experimental scheme

The purpose of the radial impact compression test of circular granite under the condition of the heat treatment and water curing is to reveal the damage and failure mechanism of the rock surrounding wellbores under the dynamic disturbance of drilling and seismic waves when geothermal energy is mined in deep wells. Considering that horizontal stress is the main factor leading to the failure of the rock surrounding a wellbore, the wellbore section, rock temperature, water temperature and cycle heat time were chosen as the main factors to study regarding the damage failure mechanism of rocks surrounding wellbores. In the experiment, high-temperature-treated granite, the inner circle of circular specimens, the curing water temperature, the cycle times from heating to curing and the radical impact load were used to simulate high-temperature rocks, wellbore sections, medium-temperature recharge water, the cycle heat times and the dynamic influence of drilling and vibration, respectively, during the exploitation of geothermal energy. In the high-temperature treatment, specimens were heated at a rate of 2°C/min until the temperature reached a set temperature, and then, the temperature was kept constant for 2 hours. In water curing, high-temperature specimens were cooled to a set temperature under natural conditions, and then, the specimens were immersed in water at the same temperature for 1 hour. One cycle of heat recovery was defined as one high-temperature treatment and one water temperature curing. There were four factors in the experiment. If every factor was taken into account in the five-level test, the experimental data were too large. Considering the project overview of geothermal energy exploitation, a set of typical experimental schemes was adopted to carry out the test. At the same time, in order to ensure that the circular granite specimens produced macroscopic failure under the action of one impact load, the maximum impact pressure corresponding to the impact load that the Brazilian disk can bear at room temperature is determined as the test pressure, that is, the impact pressure is 0.2MPa. The scheme is shown in Table 1.

**Table 1:** Radial impact test scheme of circular granite under the condition of the heat treatment and water curing.

Sample number	Outside diameter/mm	Thickness/mm	Density/(g/cm^3^)	Four factor level			
				Inside diameter/mm	High-temperature treatment/°C	Curing temperature/°C	Cycles/times
GD1-1	49.88	30.48	2.60	0.00	400	40	1
GD1-2	49.79	30.93	2.61	6.25			
GD1-3	49.41	30.36	2.62	11.71			
GD1-4	49.17	30.08	2.57	17.53			
GD1-5	49.15	30.32	2.58	22.39			
GD2-1	49.80	30.11	2.51	17.82	100	40	1
GD2-2	49.88	29.86	2.58	17.93	250		
GD2-3	49.17	30.08	2.57	17.53	400		
GD2-4	49.35	30.26	2.57	17.79	550		
GD2-5	49.29	30.05	2.57	17.67	700		
GD3-1	49.86	30.13	2.61	17.97	400	10	1
GD3-2	49.27	30.23	2.58	17.48		25	
GD3-3	49.17	30.08	2.57	17.53		40	
GD3-4	49.17	30.00	2.62	18.25		55	
GD3-5	49.15	30.13	2.63	18.31		70	
GD4-1	49.17	30.08	2.57	17.53	400	40	1
GD4-2	49.09	30.17	2.63	18.08			3
GD4-3	49.17	29.95	2.61	17.74			5
GD4-4	49.15	29.81	2.63	18.39			7
GD4-5	49.00	30.31	2.63	18.01			9

### 2.4 Experimental principle

**Fig 2:**
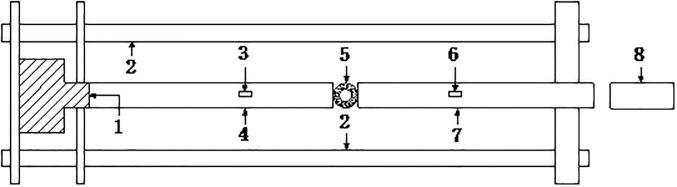
Impact loading diagram of the SHPB test system. 1-Amortisseur; 2-Support; 3-Strain gauge A2; 4-Transmission bar; 5-Rock specimen; 6- Strain gauge A1; 7-Incident bar; and8- Impact hammer.

The experiment to determine the impact compression in the heat treatment and water curing conditions was conducted on the SHPB system. The impact loading method is shown in Fig 2.

**Fig 3:**
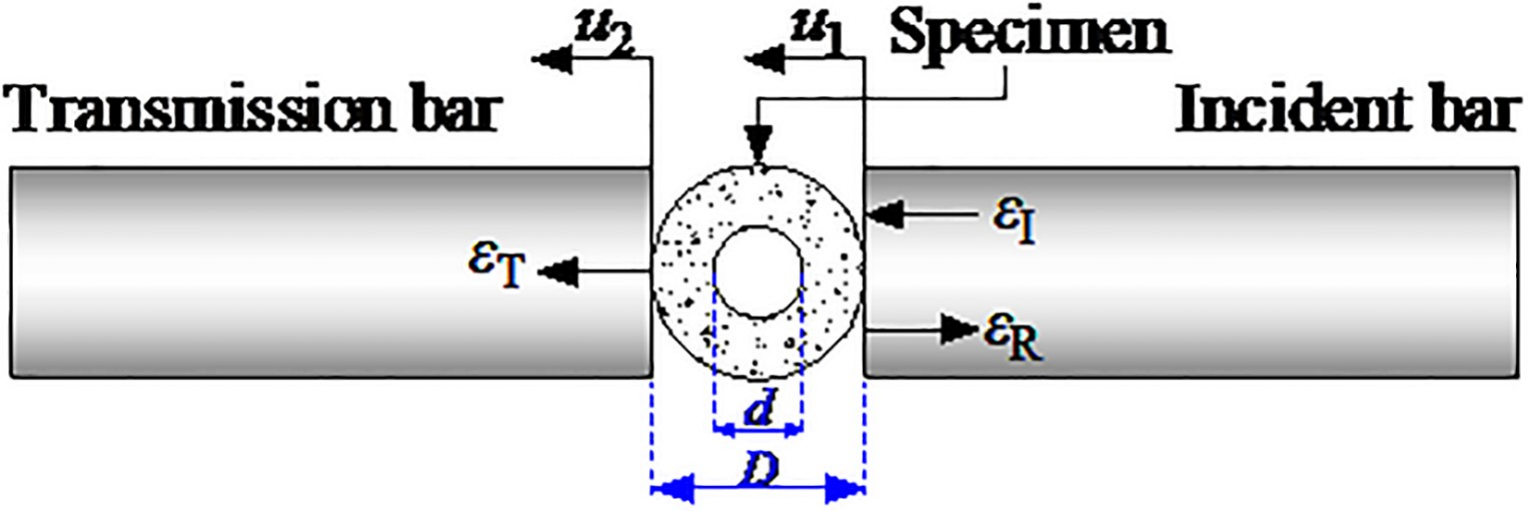
Schematic diagram of the stress deformation of circular granite under radial impact loading.

The schematic diagram of the stress deformation of the circular granite under the impact load is shown in Fig 3.

In Fig 3, platen 1 refers to the contact surfaces between the incident bar and circular specimen, platen 2 refers to the contact surface between the transmitted bar and circular specimen, and *ε*_I_, *ε*_*R*_, and *ε*_T_ are the dynamic strains of the incident bar, reflected bar and transmitted bar, respectively. According to the one-dimensional elastic wave theory and considering the time of impact loading at the same time, the impact loads of both platens can be obtained as follows:

$$F_{1}\left(t\right)=EA\left[\varepsilon_{I}\left(t\right)-\varepsilon_{R}\left(t\right)\right]$$ (1)

$$F_{2}\left(t\right)=EA\varepsilon_{T}\left(t\right)$$ (2)

The average impact load on both sides of the circular granite can be expressed by the following equation:

$$F\left(t\right)=\frac{F_{1}\left(t\right)+F_{2}\left(t\right)}{2}=\frac{EA\left[\varepsilon_{I}\left(t\right)-\varepsilon_{R}\left(t\right)+\varepsilon_{T}\left(t\right)\right]}{2}$$ (3)

Based on the fact that a semi-sine wave was used to place the load on the rock samples with the same diameter of the SHPB elastic bar, the two ends of the rock samples could reach a state of force equilibrium. Therefore, the balance hypothesis of the following equation can be introduced.

$$\varepsilon_{T}\left(t\right)-\varepsilon_{I}\left(t\right)+\varepsilon_{R}\left(t\right)=0$$ (4)

By substituting formula (4) into formula (3), the impact load that the circular granite specimen could bear in the process of impact loading can be obtained by formula (5).

$$F\left(t\right)=EA\varepsilon_{T}\left(t\right)$$ (5)

In formula (1) to (5), *E* is the elastic modulus of the incident bar and the transmission bar, and *A* is the cross-sectional area of the incident bar and the transmission bar.

## 3 Experimental results

### 3.1 Dynamic deformation characteristics

**Fig 4:**
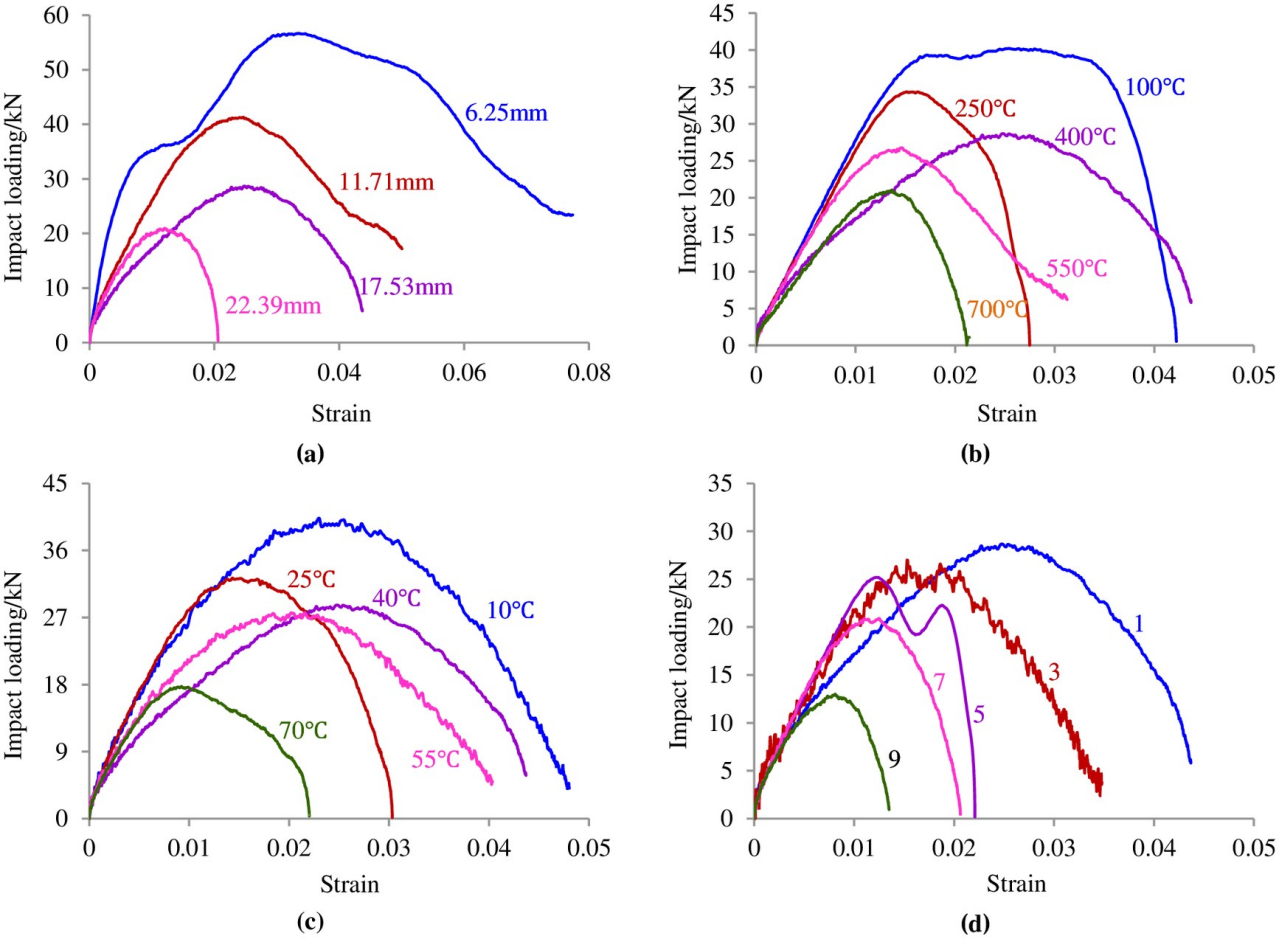
Impact load-displacement curves of circular granite samples under the condition of the heat treatment and water curing. **(a)** Load-strain curves of circular rock samples with different inner diameters (The figures in the figure represent the inner diameter of the circular granite specimens). **(b)** Load-strain curves of circular rock samples under different high temperatures (The figures in the figure represent the heat treatment temperature). **(c)** Load-strain curves of circular rock samples different curing temperatures (The figures in the figure represent the curing water temperature). **(d)** Load-strain curves of circular rock samples with different times of heating (The figures in the figure represent the number of cyclic impacts).

During the exploitation of geothermal energy, the rock surrounding a high-depth wellbore is not only in the environment of the heat treatment and water curing but is also affected by thermal impacts, earthquake wave impacts, and drilling vibrations. Based on the engineering environment of the rock surrounding a high-depth wellbore, an experiment inducing a radical impact in the heat treatment and water curing conditions was carried out to investigate the deformation characteristics of the circular granite. The typical impact load-strain curves of the circular granite with different inner diameters, high temperatures, curing temperatures and cycle heat times in the heat treatment and water curing conditions are shown in Fig 4.

Fig 4 shows that the impact load-strain curve of the circular granite samples in the heat treatment and water curing conditions changed non-linearly in general, indicating that the influence of the inner diameter, heating temperature, curing temperature and number of heat cycles on the deformation characteristics of circular granite was rather minor. To further analyse the impact load-strain curve, it can be divided into three stages: the straight stage, yield stage and post-peak stage. The initial stage of the curve is a straight stage, indicating that elastic deformation is first generated in the circular specimen under the impact load. Since the effect of stress on the inner hole wall was greater under the impact load, at the moment of impact, the internal micro-cracks of the circular granite were unable to close, and micro-cracks were generated in the whole ring structure along the loading direction, which made the initial stage of the impact load-strain curves become a straight stage but not an upper concave stage. After elastic deformation, the damage stage is generated in the circular granite. Along the direction of the impact load, the newly generated cracks are first connected, and then, the newly generated cracks in the direction of the vertical impact load are connected, which is shown as a nonlinear ascension in the impact load-strain curve. When the impact load arrived at the peak value that the circular granite could bear, macro-failure was generated in the circular granite. Due to the short impact load time, the toughness of the ring structure could defer the partial impact load effect to decrease the brittleness and strengthen the plasticity, which is shown as a non-linear drop (not a cliff-like drop) in the impact load-strain curve.

Fig 4 also shows that the impact load-strain curve under part of the factors is distorted. For example, in Fig 4a, when the inner diameter of the circular granite is 6.25 mm, the platform segment appears before the curve peak. It is deduced that the crack propagated along the impact direction under the impact load and that a crack fracture was generated. The circular specimen developed in the trend of breaking into two half rings in the direction of the impact load. During this period, the contact surfaces among the circular specimens, incident bar and transmitted bar transformed from lines to surfaces; the contact spaces became greater; and the radical strain suddenly increased, leading to a platform stage before reaching the peak value in the impact-strain curve. In Fig 4d, two peak values appeared at a heat cycle time of 5 in the curve because the circular specimen was first split into two fragments and the two half rings did not leave the incident bar and transmitted bar in time during the impact load. In this period, the impact load was supported by the two half rings, and the second peak load appeared. Because the inner damage of the whole-circular granite specimen is far smaller than that of the two-half-circular granite, the second peak load was smaller than the first peak load.

### 3.2 Change in dynamic peak load

The maximum impact load that the circular granite could bear under radical impact loads in the heat treatment and water curing conditions could reflect the ability to resist the impact of the ring structure. The structural strength of the circular specimen was calculated based on elasticity mechanics. Currently, the Brazilian strength formula and Hobbs strength formula for astatic load are generally acknowledged. To investigate the ability to resist the impact of circular specimens in the heat treatment and water curing conditions, the formula for calculating the tensile strength of Brazilian discs and rings was introduced to calculate the dynamic tensile strength of circular granite samples, and the calculation formula adopted is as follows:

$$\sigma_{1}{}_{\text{d}}=\frac{2F}{\pi Dh}\;(\text{Brazil}\;\text{strength})$$ (6)

$$\sigma_{2}{}_{\text{d}}=\frac{2F}{\pi Dh}(6\text{+}\frac{\text{38}r^{2}}{R^{2}})\;(\text{Hobbs}\;\text{strength})$$ (7)

Where *D* is the diameter of the circular specimen, *h* is the height of the circular specimen, and *R* and *r* are the radii of the circular specimen and inner ring, respectively.

**Fig 5:**
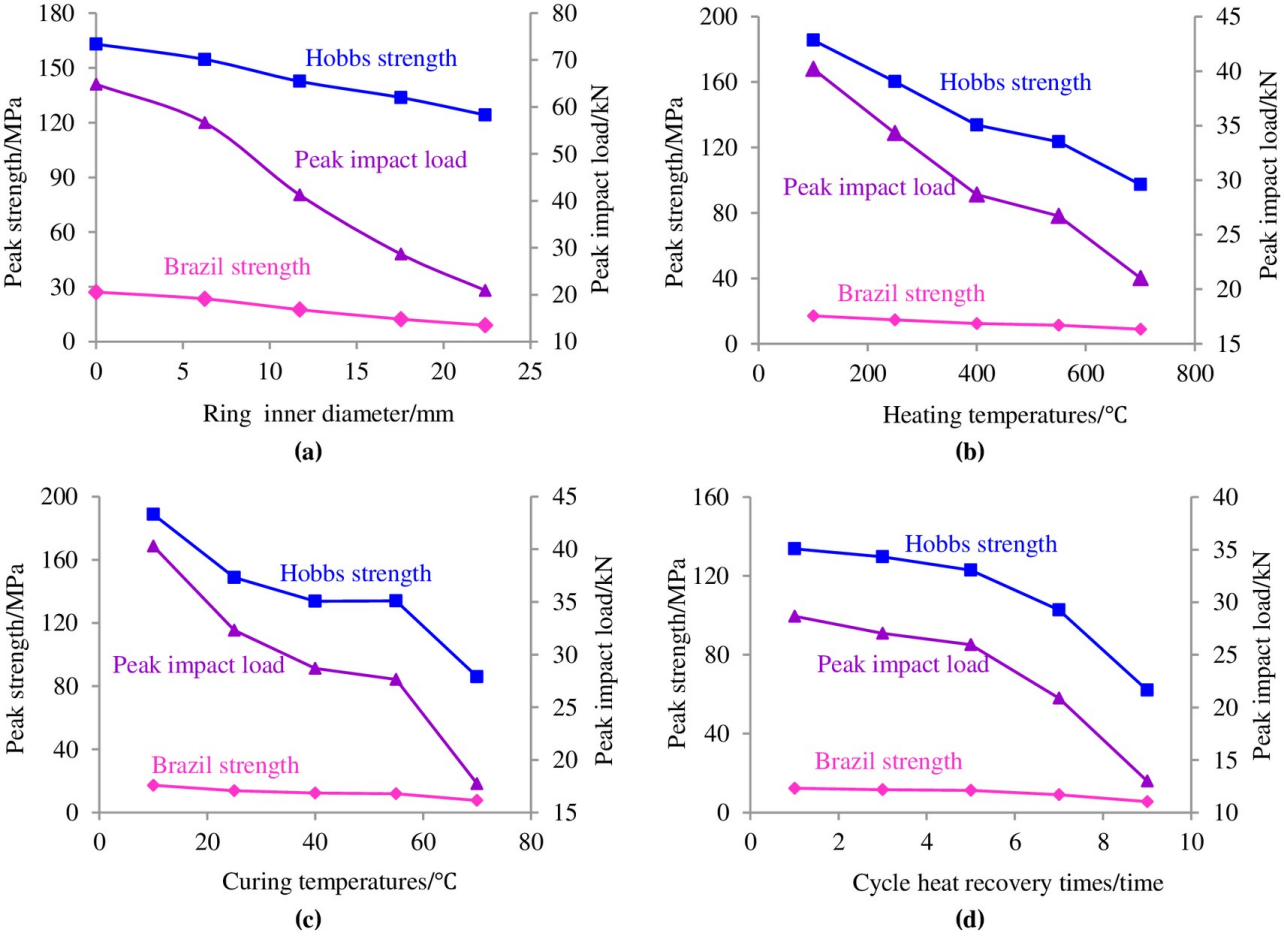
Changing relationships of the Hobbs intensity, Brazil strength and peak impact load. **(a)** Different ring inner diameter. **(b)** Different heating temperatures. **(c)** Different curing temperatures. **(d)** Different cycle heat recovery times.

The changes in the Brazilian tensile strength, Hobbs tensile strength and peak impact load as the inner diameter of the ring, heat temperature, curing temperature and cycle heat time change are shown in Fig 5.

In Fig 5, the Hobbs tensile strength, peak impact load and Brazil tensile strength decreased as the inner diameter of the circular granite, heat temperature, curing temperature and cycle heat recovery time increased. The Hobbs tensile strength was far greater than the Brazil tensile strength under the same conditions. The Hobbs tensile strength and Brazil tensile strength were calculated based on the elasticity, and the influence of the ring inner diameter was not considered in the calculation of the Brazil strength; however, the influence of the ring inner diameter was considered in the Hobbs strength. The inner circle of circular granite causes an obvious stress concentration on the ring wall, and as a result, the Hobbs strength is greater than the Brazil strength, which can also be obtained from the formulas used to calculate the Hobbs strength and Brazil strength. With the increase in the inner diameter, the dynamic tensile stress in the ring wall along the loading direction became rather noticeable, and the compressive stress perpendicular to the loading direction also became significant. Under the combined action of the above two factors, the failure speed of the circular granite structure is accelerated, and its resistance to a dynamic impact load is noticeably weakened. Fig 5a shows that the Hobbs tensile strength, peak impact load and Brazil tensile strength decreased under this condition. When the circular granite was treated at a high temperature, the moisture in the inner structure gradually evaporated with increasing temperature and the porosity inside the specimen increased. As the temperature became high, the expansion of rock particles after thermal treatment became rather noticeable and the thermal stress of the structure among the particles strengthened. Under this condition, the generation of new micro-cracks was promoted and the difficulty of tensile cracks penetrating along the impact direction and the vertical impact direction was also reduced. Ultimately, the capacity of the circular granite to bear the radical impact load weakened, which can be seen in Fig 5b. When the circular granite after high-temperature treatment was placed in water, the water molecules entered the pores in the rock sample. As the water temperature increased, the mobility of the water molecules became stronger, and they penetrated faster into the micro-cracks in the circular granite. For a constant curing time, the extent of the water saturation of the circular granite increased as the curing temperature increased. In turn, the capacity to bear the radical impact load weakened, as shown in Fig 5c. The capacity to resist the impact load weakened as the cycle heat time increased, as shown in Fig 5d. This result can be explained by the cycle influence from high time treatment to warm-water curing; the circular granite was in the cycle condition, including dehydration and saturation. At the same time, the structural thermal stress between the rock particles was also in a cyclic state of enhancement and weakening. With each cycle, the circular granite incurred some damage, and the extent of the accumulative failure damage became serious with increasing cycle times.

### 3.3 Change in the inner hole strain

**Fig 6:**
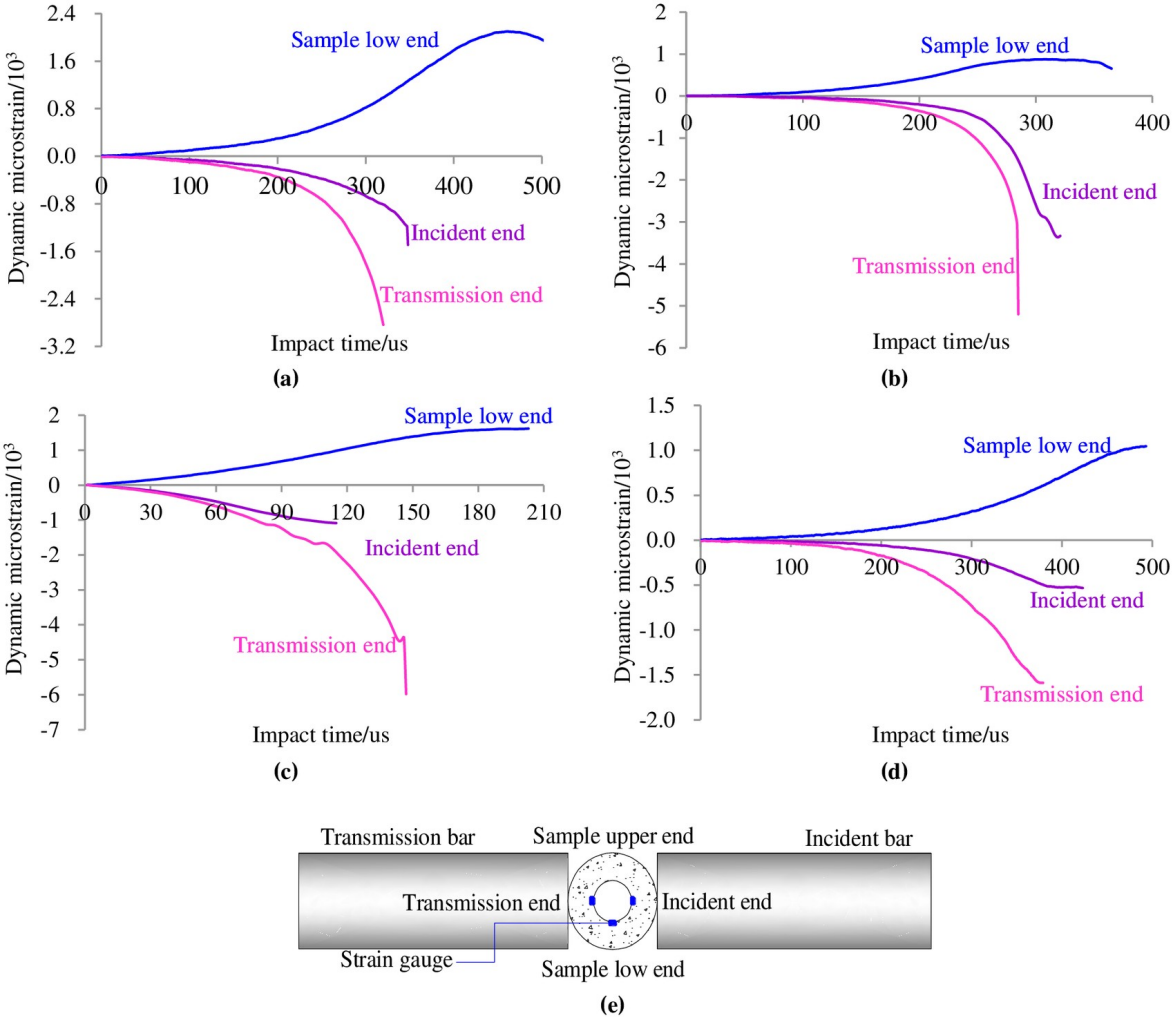
Changes in the ring wall dynamic strain at the incident end, transmission end and lower end of circular granite with loading time. **(a)** Sample GD1-3. **(b)** Sample GD2-2. **(c)** Sample GD3-2. **(d)** Sample GD4-5. **(e)** Schematic diagram of position marking of rock sample.

The most direct manifestation of high-depth wellbore failure is that the wall tension or compression displacement exceeds the limit of the rock surrounding the wellbore. By analysing the change in the dynamic strain in the ring wall as the loading time increased in the heat treatment and water curing conditions, the mechanism of damage failure of the circular granite can be revealed, which can provide a reference for further investigating the mechanism by which a high-depth wellbore suffers deformation failure caused by impact disturbance. Fig 6 shows the change in dynamic strain at the incident end, transmission end and lower end of the circular granite with loading time under four groups of different influencing factors.

The figure shows that the strain at the incident end, transmitted end and low end increased with the impact loading time. The tensile strain perpendicular to the impact direction was generated at the incident end and transmitted end of the ring wall, while the compressive strain parallel to the loading line was generated at the low end. Comparing the tensile strain and compressive strain, it is found that the difference between the two values is not large. Since the compressive capacity of the rock is much higher than its tensile capacity, tensile failure is first generated along the impact loading direction, and the dynamic tensile strain effect perpendicular to the impact loading direction is the most direct factor leading to the instability of the circular granite structure.

The figure also shows that as the inner diameter of the circular granite, heat temperature, curing temperature and cycle heat times changed, the maximum compressive strain at the low end lagged behind the maximum tensile strains at the incident end and transmitted end. The failure of the circular granite at the low end lagged behind the failures of the circular granite at the incident end and transmitted end, which indicated that the micro-fracture surface was generated along the impact direction first, and then, the penetrated fracture surface was generated perpendicular to the impact loading direction under the impact load. The tensile strain of the ring wall at the transmitted end was larger than the tensile strain at the incident end, and the tensile strain at the transmission end also reached the maximum in advance. This phenomenon indicates that the tensile failure was first generated along the impact loading direction at the transmitted end in the specimen and that the failure at the incident end lagged behind the failure at the transmitted end. When the incident end of the ring wall was affected by the impact stress wave, the incident stress wave and the reflected stress wave were accompanied. The incident stress wave belonged to the compressive wave, and the transmitted stress wave belonged to the tensile wave. The above two waves acted together on the incident end of the ring wall, delaying the generation, propagation and penetration of the tensile micro-crack; however, the transmission wave generated at the transmission end was a tensile stress wave, which promoted the generation, propagation and even penetration of the tensile crack.

### 3.4 Damage failure characteristics

#### 3.4.1 Damage evolution process

**Fig 7:**
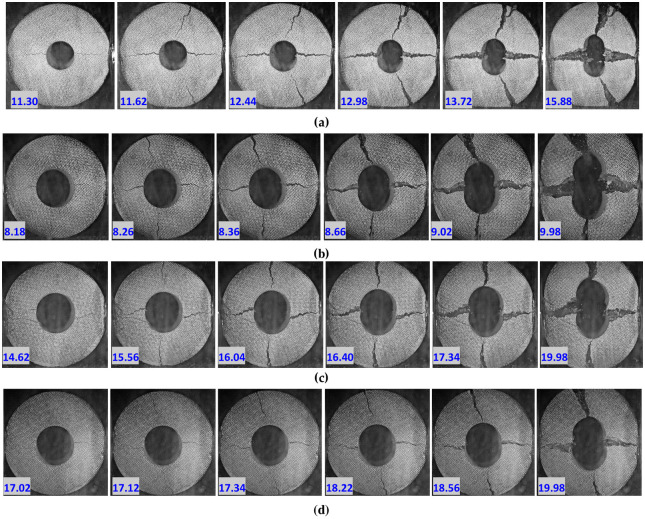
Damage history process of circular granite under a radial impact load (the number in the figure represents the moment when the camera took the photo; for example, 11.3 represents the time of 11.3 ms when the shooting started). **(a)** Sample GD1-3. **(b)** Sample GD2-2. **(c)** Sample GD3-4. **(d)** Sample GD4-2.

The failure mechanism of the circular granite was also revealed by analysing the damage failure evolution process of the circular granite under the impact load. Fig 7 shows 4 groups of typical crack propagation processes of the circular granite under different environmental conditions.

Fig 7 shows that under the radical impact load, a crack initiates in, expands in and penetrates the circular granite, and the fracture surface begins to be generated. The crack starts from the inner diameter end of the ring and gradually expands to the outer side of the ring. It is found from the crack propagation process that the crack spacing is gradually enlarged, and no dislocation occurs between the two failure surfaces of the crack. The crack is a dynamic tensile crack, which means that the dynamic tensile effect appears perpendicular to the impact direction, and the effect of dynamic tensile stress is most obvious at the inner diameter of the ring, where the fracture first occurs. The fracture surface perpendicular to the impact loading starts to generate from the outer ring to the inner diameter. The two fracture surfaces of the crack are not displaced, indicating that the failure is a tensile failure. According to elasticity theory, the mechanical effect in the inner hole of the circular granite is larger than that in the outer ring wall. A compressive stress effect is generated in the inner hole of the circular granite, while a tensile stress effect is generated in the outer ring. Since the compressive strength is larger than the tensile strength for the rock mass, the fracture surface expands from the outer ring to the inner hole.

In Fig 7, the left side of the rock sample is the incident bar and the right side is the transmission bar. Analysing the crack pattern along the impact loading line at the two sides of the inner hole in the circular granite, it is found that the crack in the transmitted bar was generated earlier than that in the incident bar and that the micro-fracture surface was first generated on the right side of the inner ring wall, which can be seen clearly at 17.2 ms in Fig 7d. At the same time, it is also found that the cracks on the side of the transmission bar extend rapidly, the separation distance between the cracks is larger, and the fracture surface penetrates earlier. To better illustrate the reason for these results, it can be seen that the incident stress wave travels through the circular granite, part of the wave is reflected by the inner hole, the dynamic tensile effect generated by the incident stress wave is offset, and the crack generation and the extension of the inner hole on the side of the incident bar are delayed. However, the transmission of the stress wave is a tensile stress wave, and the tensile stress effect generated on the side of the transmitted bar is more apparent. The inner hole wall is first split and is then gradually penetrated on the side of the transmitted bar.

**Fig 8:**
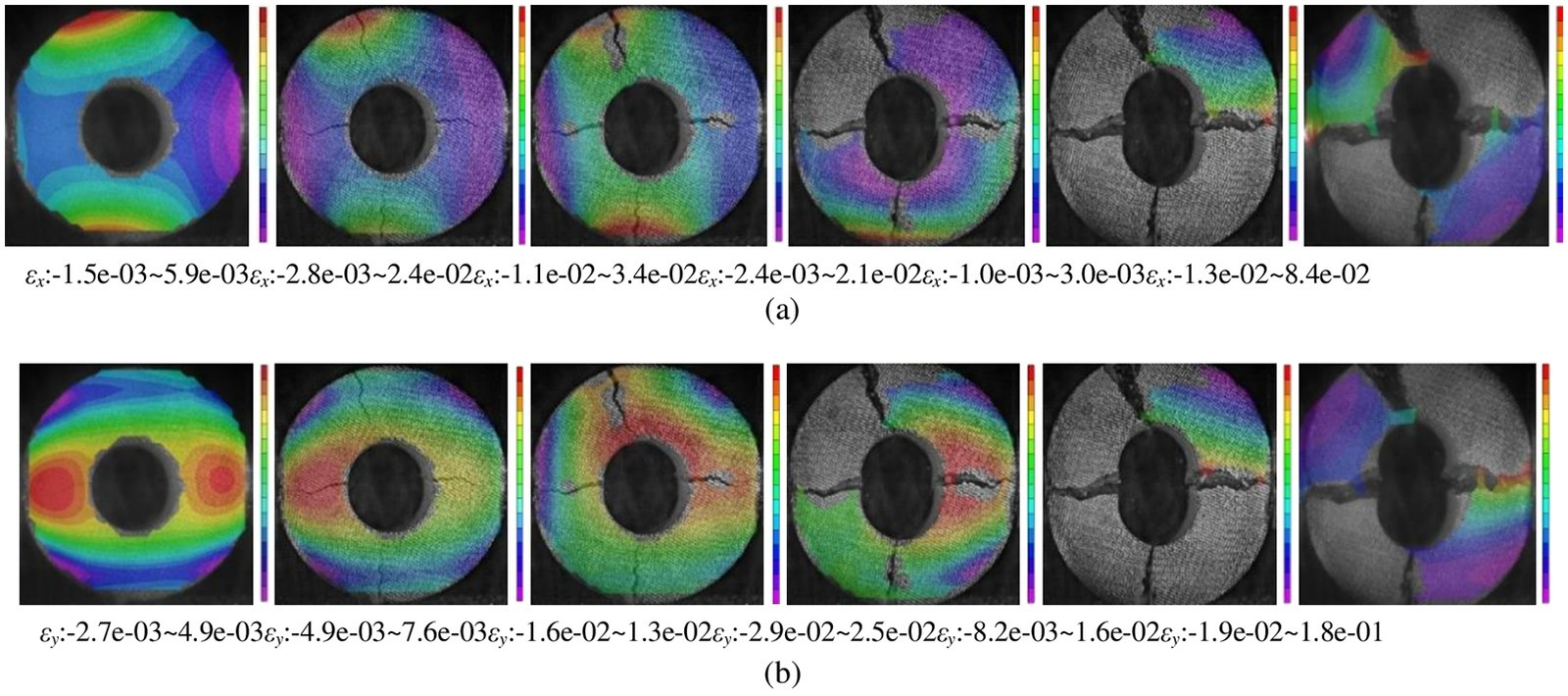
Strain history of the circular granite under radial impact load (sample GD2-2). (a) Evolution of the strain cloud image in the x direction (the direction of the impact stress wave propagation is the x direction). (b) Evolution of the strain cloud image in the y direction (the direction of vertical impact stress wave propagation is the y direction).

Using VIC-3D non-contact digital speckle technology to analyse the lateral strain evolution of the circular granite under a radial impact load, the damage degree of the circular granite at each position can be effectively revealed. The result is shown in Fig 8.

In Fig 8, the strain along the travelling direction of the impact stress wave is larger, and the strain before the crack extends is larger than that after the crack extends. The strain in the outer ring perpendicular to the travelling direction of the impact stress wave is larger, and the value decreases with the decrease in the radius of the ring. It is shown again that the circular granite is first split and penetrated from the inner hole to the outer ring along the impact direction, and the circular specimen is split and penetrated from the outer ring to the inner wall vertical to the impact direction. The cloud maps of strain along the travelling direction of the impact stress wave are shown in Fig 8a. The picture clearly shows that the tensile strain perpendicular to the impact direction in the outer ring is larger, indicating that the crack perpendicular to the impact direction is determined by the tensile stress effect parallel to the impact direction, which is generated by the impact stress wave. The cloud maps of the strain perpendicular to the travelling direction of the impact stress wave in Fig 8b show that the crack split along the impact direction is determined by the tensile stress perpendicular to the impact direction generated by the impact stress wave. Based on the analysis of the strain cloud maps in the x direction and y direction, it can be verified that tensile failure is generated in the circular granite under the radical impact load.

#### 3.4.2 Failure modes

**Fig 9:**
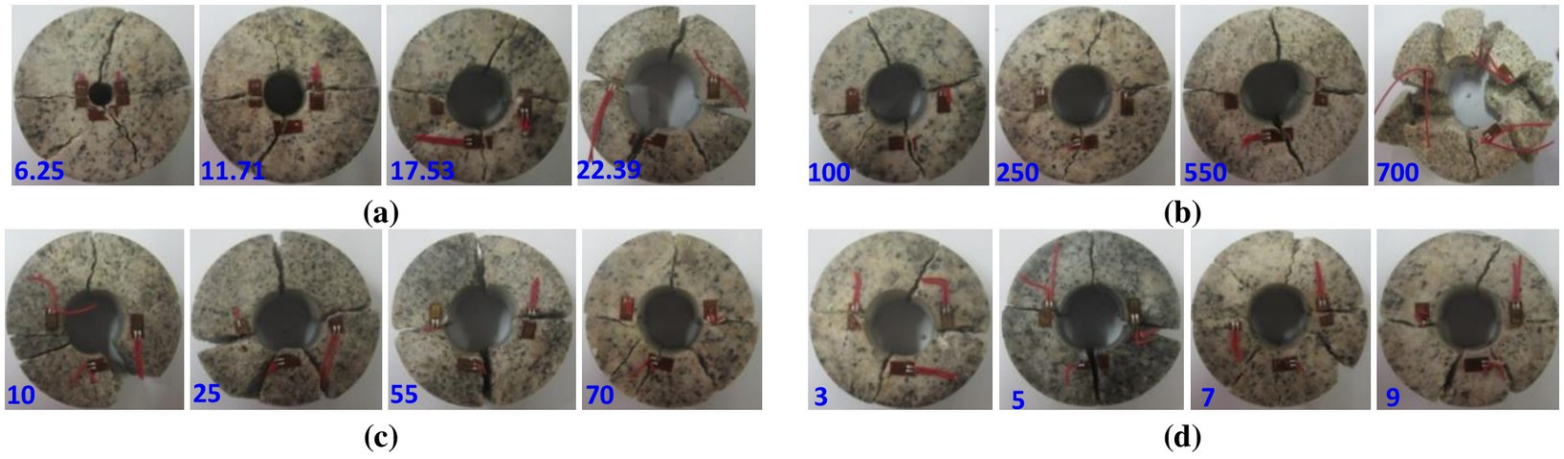
Failure modes of circular granite under radial impact loads. **(a)** Different ring inner diameters (Units: mm). **(b)** Different heating temperatures (Units: °C). **(c)** Different curing temperature**s** (Units: °C). **(d)** Different cycle heat recovery times (Units: time).

The failure modes can reveal the failure mechanism of the circular granite under theradical impact load in the heat treatment and water curing conditions. The final failure modes of the circular granite specimens with different inner diameters, heat temperatures, curing temperatures and cycle heat times under the radical load are shown in Fig 9.

Analysing the failure modes of each ring specimen shown in Fig 9, it is found that two principal fracture zones are generated in the circular specimen. One fracture surface is formed when the cracks penetrate along the impact load direction, and the other fracture surface is formed when cracks penetrate along the direction that is intersected by the impact direction. The circular granite is split after the penetration of the two fracture surfaces. The occurrence of the two main crack zones reveals that there are two concentration zones of the tensile stress effect in the circular granite under the radial impact load, which are the main factors leading to the failure of the ring sample. After further analysing the failure fragments of the circular specimens, it is found that the split blocks of the circular specimens increase with larger inner diameters, higher heating temperatures, higher curing temperatures, and more cycle heat times, indicating that the failure degree of the circular granite is more serious. The block after the splitting test shows that the fracture surface is smooth and that there is no trace of friction dislocation. Then, it is found that the broken specimens can return to the complete ring structure along the joint. The fracture surfaces are better coupled, which again shows that tensile failure is generated in the circular granite under impact load, and the ring inner diameter, heating temperature, curing temperature and cycle heating time are of less significance to the failure mode of the circular rock specimens.

## 4 Failure criterion of the circular granite

### 4.1 Assumptions

As rock is the product of geological processes, it is discontinuous, anisotropic and non-uniform, which makes study of the failure criterion of rock difficult. As a result, the derivation of the rock failure criterion needs to be based on certain assumptions. Based on the deformation characteristics and failure characteristics of circular specimens under the radical impact load in in the heat treatment and water curing conditions, the following assumptions are proposed as the basis for the failure criterion of circular granite.

(1) Since a micro-fracture is generated suddenly in the circular granite under the impact load, it is assumed that the cross-sectional area of the circular granite before the impact failure remains constant.
(2) It is assumed that the circular granite has elastic characteristics, and its mechanical parameters can be derived by an elastic formula.
(3) The direct factor of the failure of the circular granite under the radial impact load is the tensile stress effect along the impact direction and the vertical impact direction. Therefore, Hooke’s law is assumed to govern the relationship between the tensile stress and tensile strain.
(4) Under the impact load, the rock specimen is deformed in an elliptical trend, and the deformation and strain in the inner ellipse of the circular specimens are even.
(5) When the radical impact load that the circular granite can bear increases to the peak value, it is considered that the circular specimen has been broken at that moment.

### 4.2 Deduction of failure criterion

**Fig 10:**
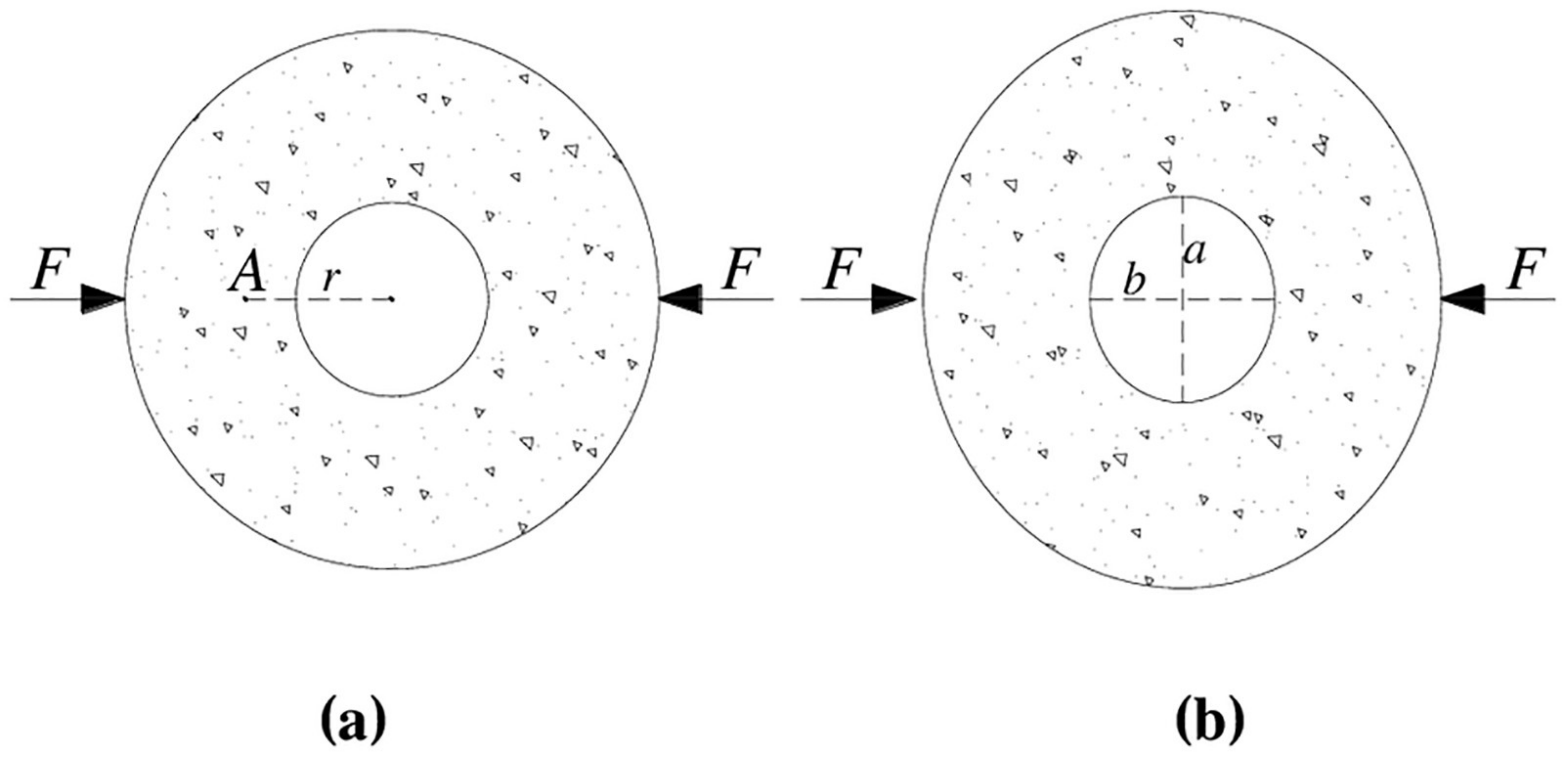
Schematic diagram of the impact compression deformation of circular granite. **(a)** Before impact deformation. **(b)** After impact deformation. (In the figure, F is the radial impact load, A is any point in the ring, r is the circle radius of the small circle inside the ring, and a and b are the long and short semi-axial lengths of the ring after deformation, respectively).

Based on assumptions (1)—(5), elasticity theory is used to deduce the dynamic strain at any point of the circular granite. By comparing the strain with the maximum dynamic tensile strain that the circular granite can bear, the failure criterion of the circular granite under the radical impact load can be established. The dynamic deformation of circular specimens under the radical impact load is shown in Fig 10.

Based on assumptions (1) and (4), the cross-sectional area of the circular granite after impact compressive deformation remains constant. The cross section of the rock sample changes from circular to elliptic, and the corresponding area calculation formula can be obtained as follows:

$$S=\pi r^{2}=\pi a\left(r-\frac{u}{2}\right)$$ (8)

where *S* is the cross-sectional area of the surface across point *A*, *u* is the impact compressive displacement, *a* is the long half axis of the ellipse across point *A* after the impact deformation of the circular granite, and *r* is the radius between point *A* and the centre of the inner circle.

Based on assumptions (3) and (4), the amount of dynamic deformation of the circle across point *A* can be considered to be the value of the ellipse circumference across point A and concentric with the inner ellipse after the impact compressive deformation subtracts the circle circumference across point *A* before the impact compressive deformation. Therefore, the strain at point *A* after the impact load can be calculated by the following equation.

$$\varepsilon_{A}=\frac{L_{2}-L_{1}}{L_{1}}$$ (9)

Where *ε*_*A*_ is the strain at point *A*, *L*_1_ is the circumference of the circle across point *A* before the impact compressive deformation, and *L*_2_ is the circumference of the ellipse across point *A* after the impact load.

According to formula (8), (9) and (10) can be obtained as follows:

$$\varepsilon_{A}=\frac{8ru-2u^{2}-2\pi ru+\pi u^{2}}{2\pi r\left(2r-u\right)}$$ (10)

Based on assumption (3), the granite specimens obey Hooke’s law. The relationship among the dynamic tensile strength (*σ*_dt_) of granite, the dynamic tensile strain *ε*_d_ and the dynamic tensile elastic modulus *E*_d_ is expressed as follows:

$$\sigma_{\text{d}}{}_{\text{t}}=E_{\text{d}}\varepsilon_{\text{d}}$$ (11)

The dynamic tensile strength of granite can be measured indirectly by the dynamic Brazilian splitting test, and its calculation formula is as follows:

$$\sigma_{\text{d}}{}_{\text{t}}=-\frac{2F_{\text{max}}}{\pi dh}$$ (12)

where *F*_*max*_ is the maximum impact load, *d* is the diameter of the Brazilian disc, and *h* is the height of the Brazilian disc.

By substituting formula (11) into formula (12), the maximum tensile strain that point *A* bears is calculated by

$$\varepsilon_{\text{dmax}}=\frac{2F_{\text{max}}}{\pi dhE_{\text{d}}}$$ (13)

By analysing the dynamic tensile strain at point *A* of the disk granite, it can be judged whether the circular granite is damaged. The criterion formula is as follows:

$$\left\{ \begin{array}{l} \frac{8ru-2u^{2}-2\pi ru+\pi u^{2}}{2\pi r\left(2r-u\right)}>\frac{2F_{\text{max}}}{\pi dhE_{\text{d}}}{}^{}\;(\text{Destroyed})\\ \frac{8ru-2u^{2}-2\pi ru+\pi u^{2}}{2\pi r\left(2r-u\right)}\leq \frac{2F_{\text{max}}}{\pi dhE_{\text{d}}}{}^{}\;(\text{Not}\;\text{destroyed}) \end{array}\right.$$ (14)

### 4.3 Determination of the failure criterion parameters

**Fig 11:**
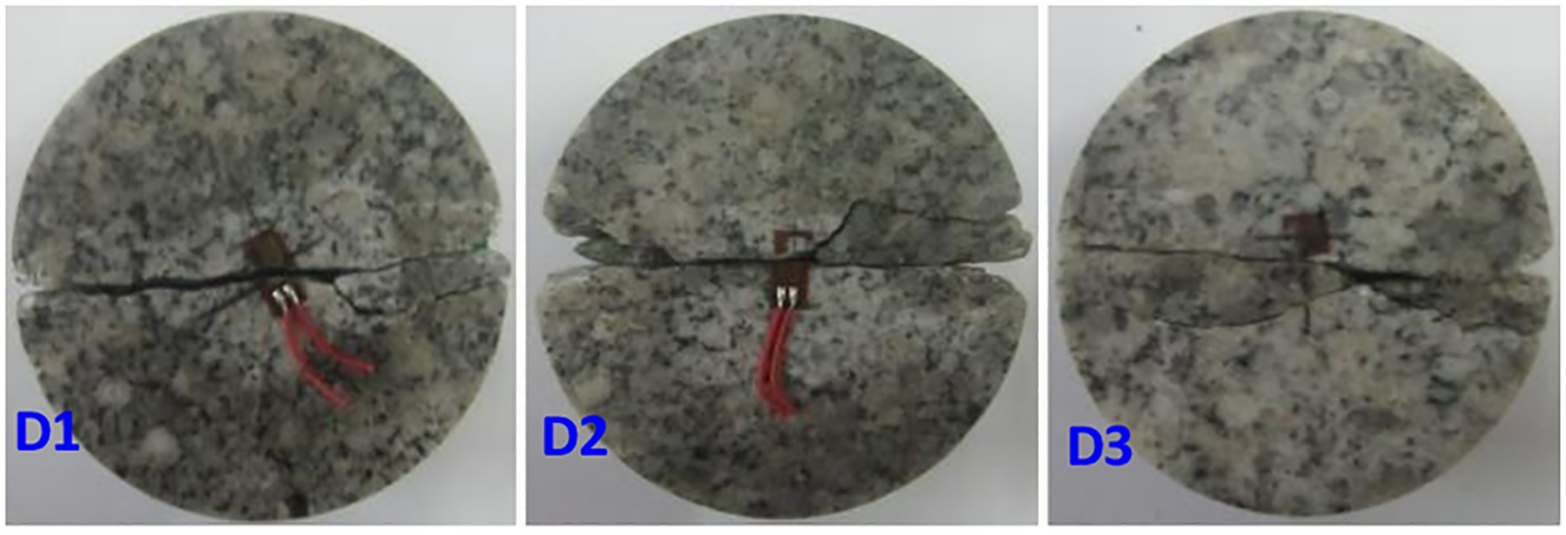
Failure model of the Brazil disk split test.

The dynamic tensile strength and dynamic tensile elastic modulus accurately measured by the test are the keys to analysing the failure criterion of the circular granite under the impact load. In the test, the strain gauge was pasted in the centre of the Brazilian splitting disk, and then, the impact load of the vertical strain gauge was applied. The strain measured by the strain gauge is considered to be the dynamic tensile strain of the disc under the impact load. When the impact load reached the maximum, the tensile stress calculated by formula (12) is considered to be the dynamic tensile strength of the circular granite. The failure mode of discs under the impact load is shown in Fig 11.

In Fig 11, the three discs are split into two fragments, and the fracture surface penetrates to the middle of the strain gauges, which means that dynamic tensile failure is generated in the circular specimen under the impact load and that the strain detected by the strain gauge is the dynamic tensile strain. Therefore, the dynamic tensile elastic modulus of the circular specimens can be calculated by Hooke’s law. The results of the failure criterion parameters detected by the test are shown in Table 2.

**Table 2:** Test results of the failure criterion parameters.

Sample number	High-temperature Treatment/°C	Curing temperature /°C	Dynamic tensile Strength/MPa	Dynamic stretch elasticity Modulus/GPa
D1	400	40	25.19	18.13
D2			26.37	20.69
D3			24.95	17.28
Average			25.50	18.70

### 4.4 Test verification

A group of circular specimens was used to verify the failure criterion of the circular specimens under the impact load. The heating temperature of the rings was 400°C, the curing temperature was 40°C, and the diameters of the specimens were 6.25 mm, 11.71 mm, 17.53 mm and 22.39 mm. Since the fracture of the circular specimens initiated from the inner wall and expanded to the outer ring along the impact loading direction under the impact load, the rationality of the failure criterion can be verified by determining whether the inner ring wall of the ring is damaged along the direction of the impact load. The verification is completed by substituting the average dynamic tensile strength and dynamic tensile elastic modulus of the specimens into formula (14), where *r* is the extreme value between the inner diameter and outer diameter of the ring. The impact compressive displacement is the displacement under the dynamic peak load and can be calculated by formula (15).

$$u=-2\int_{0}^{t}C\varepsilon_{R}dt$$ (15)

Where *ε*_*R*_ is the strain of the reflected wave detected by strain gauges on the reflected bar and *t* is the time corresponding to the maximum peak load in the process of the impact load.

**Fig 12:**
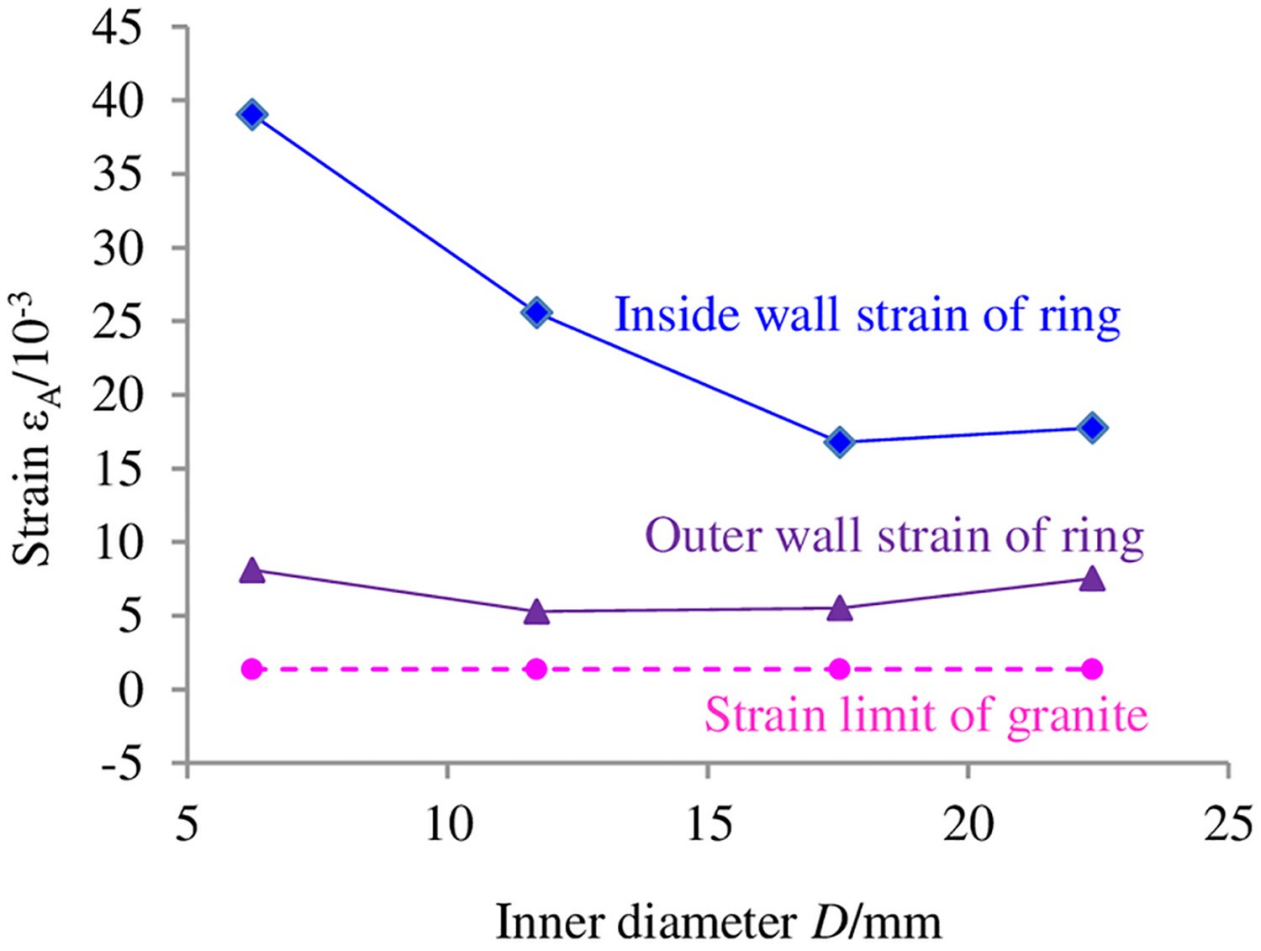
Verification result of the failure criterion test of circular granite.

The failure criterion is verified when the parameters are substituted into formula (14). The verification result is shown in Fig 12.

In Fig 12, the dynamic strain under the dynamic peak load is exported to verify the relationships between the maximum dynamic tensile strain that the circular specimens can bear and the dynamic tensile strain in the inner hole and the outer ring. The strain in the inner hole is noticeably greater than the extreme of the dynamic tensile strain in the granite specimens, and the strain in the outer ring is also more than the extreme of the dynamic tensile strain in the granite specimens but is less than the strain in the inner hole, which means that micro-fracturing has taken place in the circular specimens at that moment and that failure initiates from the inner hole of the ring. The cracks along the impact direction initiate from the inner hole and extend to the outer ring; then, perpendicular to the impact loading direction, they initiate from the outer ring and extend to the inner hole, and dynamic tensile failure is generated. The result agrees with the verification result, which indicates that the dynamic failure criterion of the circular specimens is reasonable.

## 5 Conclusions

Based on the background that the wellbore was affected by thermal impacts, earthquake wave impacts and drilling vibrations during the exploitation of geothermal energy, a study on the mechanical characteristics of circular granite under the radical impact load in the heat treatment and water curing conditions is carried out, and the conclusions are as follows:

(1) The inner diameter, heat temperature, curing temperature and cycle heat time are less significant to the deformation characteristics of the circular specimens than other parameters, and the impact load-strain curve can be divided into three stages: the initial straight stage, nonlinear ascent yield stage and post-peak nonlinear decline stage.
(2) Hobbs tensile strength, impact peak load, and Brazilian tensile strength all decrease with the increase in the inner diameters of the circular specimens, heating temperatures, curing temperatures and cycle heat times, and Hobbs tensile strength is far greater than Brazilian tensile strength, which reveals that the capacity of the circular specimens to resist the impact is mainly determined by the inner diameter of the ring, and the tensile stress concentration in the ring is more noticeable.
(3) Under the impact load, dynamic tensile failure is generated in the circular granite. The two principal fracture surfaces are generated along and perpendicular to the impact loading direction, and the cracks along the impact loading direction initiate from the inner wall. The cracks in the transmitted bar initiate earlier than the cracks in the incident bar, and the initial fractures perpendicular to the impact loading direction lag behind the other fractures. Finally, all of the cracks penetrate, which leads to micro-failure of the circular specimens.
(4) Based on the deformation and failure characteristics of the circular specimens under the impact load, the failure criterion of the maximum dynamic tensile strain in the circular specimens was established. By substituting dynamic tensile strains, tensile strengths and tensile elastic modulus detected by the Brazilian disc splitting test into the failure criterion, it is verified that the failure criterion is reasonable.

## Supporting information

S1 Data (ZIP)
