# Supplementary material for: Study on the dynamic characteristics of rock surrounding a wellbore in energy storage areas during deep geothermal energy mining
Source: PLoS One. 2020 Aug 21;15(8):e0237823. doi: 10.1371/journal.pone.0237823 (PMC7442234; doi:10.1371/journal.pone.0237823)
Supplement: S1 Data — (ZIP) [file pone.0237823.s001.zip › DATA/10+Figure 9.docx]

| 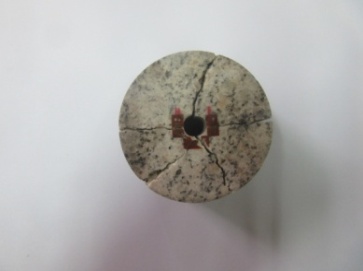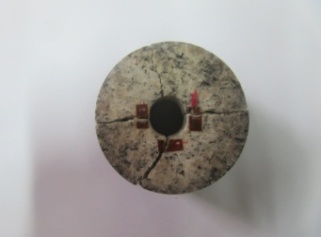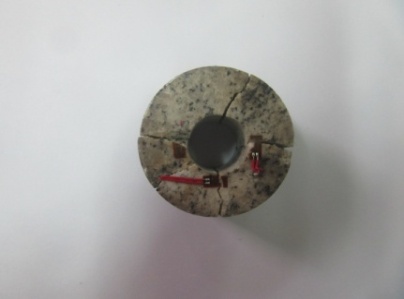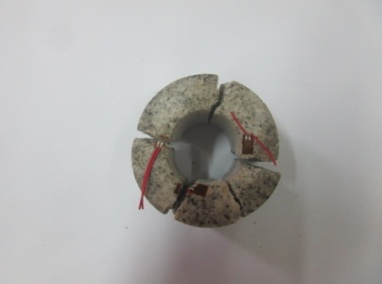  **700**  **550**  **250**  **100**  **6.25**  **22.39**  **11.71**5  **17.53**  **（a）** Different ring inner diameter  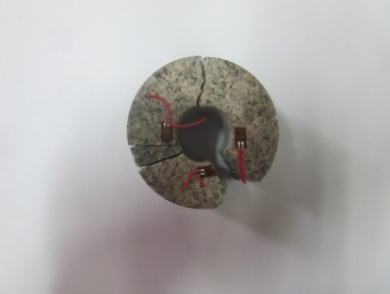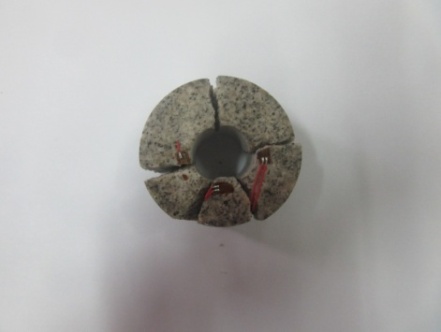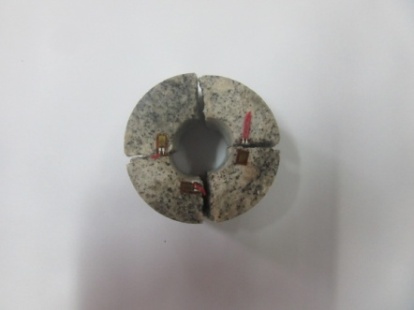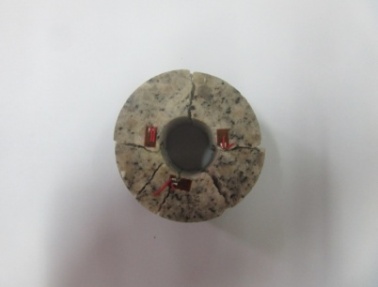  **70**  **25**  **55**  **10**  **（c）** Different curing temperature**s** | 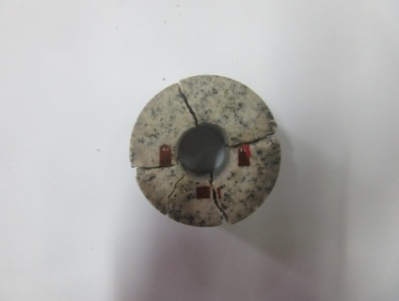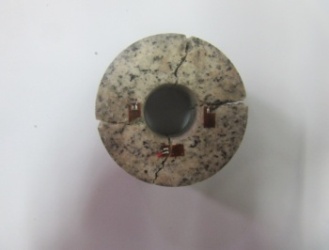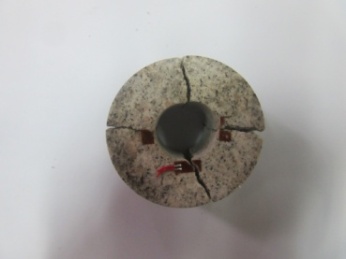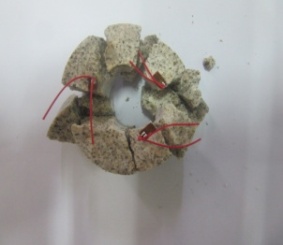  **（b）** Different heating temperatur**es**  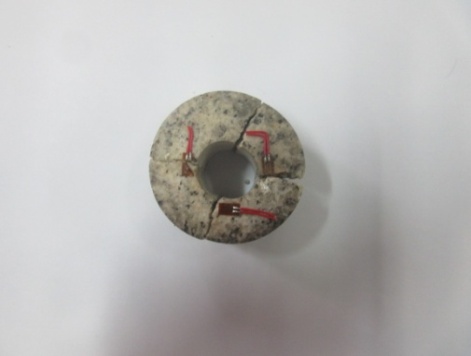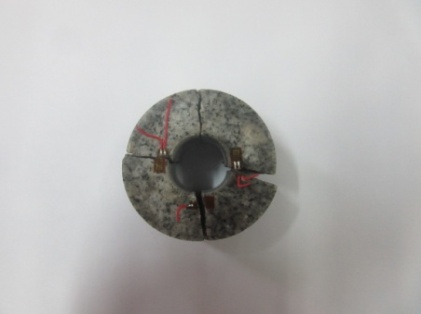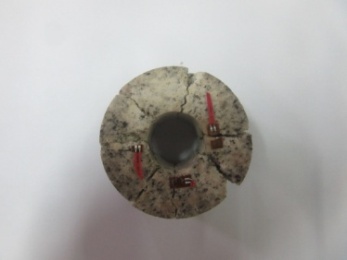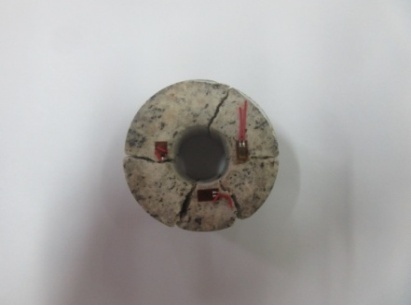  **9**  **7**  **5**  **3**  **（d）** Different cycle heat recovery times |
| --- | --- |

**Figure.9** Failure mode of ring granite under radial impact load
