# Supplementary material for: Study on the dynamic characteristics of rock surrounding a wellbore in energy storage areas during deep geothermal energy mining
Source: PLoS One. 2020 Aug 21;15(8):e0237823. doi: 10.1371/journal.pone.0237823 (PMC7442234; doi:10.1371/journal.pone.0237823)
Supplement: S1 Data — (ZIP) [file pone.0237823.s001.zip › DATA/11+Figure 10.docx]

**（a）** Before impact deformation **（b）** After impact deformation

**Figure.10** The schematic diagram of impact compression deformation of circular granite

（In the figure: F is the radial impact load; A is any point in the ring; r is the circle radius of small circle inside the ring; a, b is the long, short semi axial length of the ring after deformation, respectively）
