# Supplementary material for: Study on the dynamic characteristics of rock surrounding a wellbore in energy storage areas during deep geothermal energy mining
Source: PLoS One. 2020 Aug 21;15(8):e0237823. doi: 10.1371/journal.pone.0237823 (PMC7442234; doi:10.1371/journal.pone.0237823)
Supplement: S1 Data — (ZIP) [file pone.0237823.s001.zip › DATA/4+Figure 3.docx]

**Figure.3** Schematic diagram of stress deformation of circular granite under radial impact loading
