# Supplementary material for: Study on the dynamic characteristics of rock surrounding a wellbore in energy storage areas during deep geothermal energy mining
Source: PLoS One. 2020 Aug 21;15(8):e0237823. doi: 10.1371/journal.pone.0237823 (PMC7442234; doi:10.1371/journal.pone.0237823)
Supplement: S1 Data — (ZIP) [file pone.0237823.s001.zip › DATA/8+Figure 7.docx]

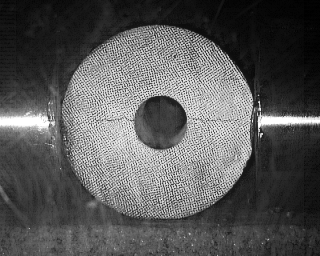

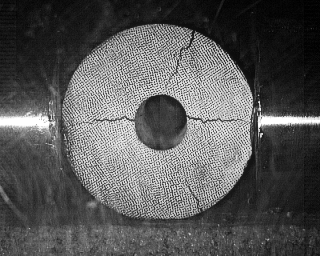

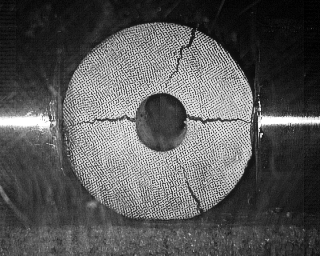

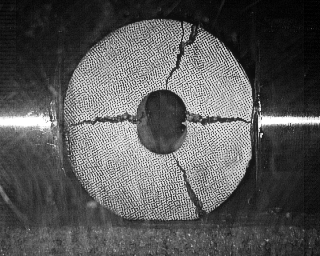
**
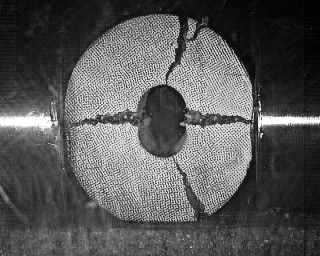
**
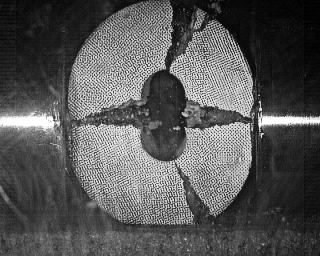


**11.30**

**11.62**

**13.72**

**12.98**

**15.88**

**12.44**

**（a）** Sample GD1-3


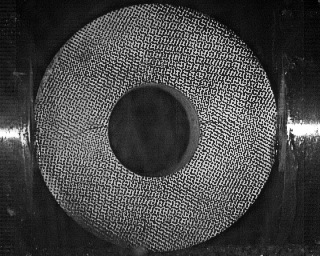

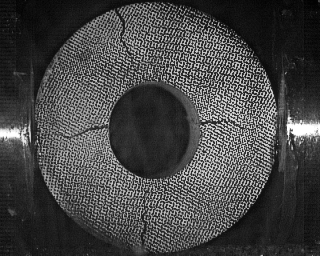

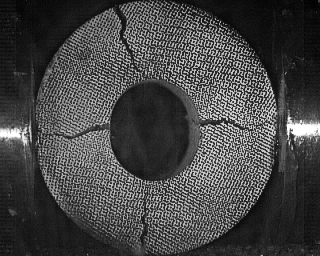

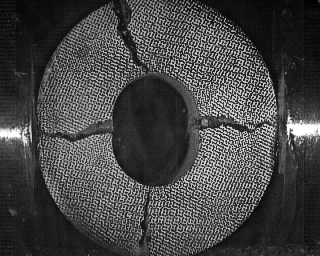

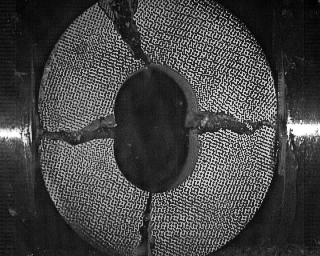

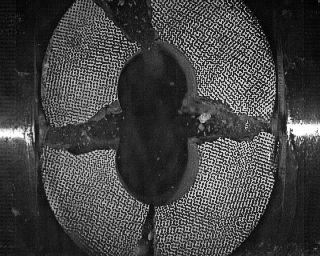


**8.18**

**8.26**

**9.02**

**8.66**

**9.98**

**8.36**

**（b）** Sample GD2-2


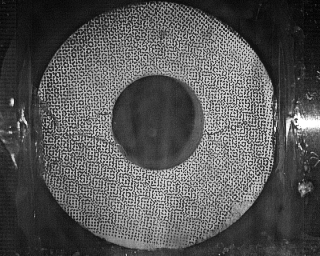

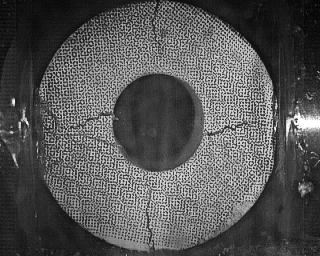

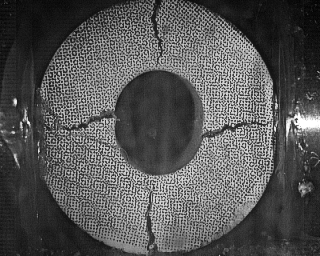

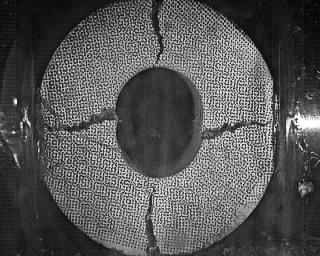

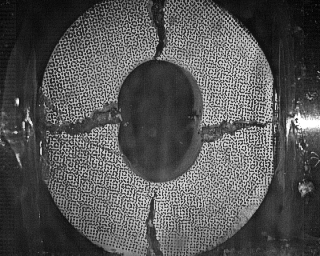

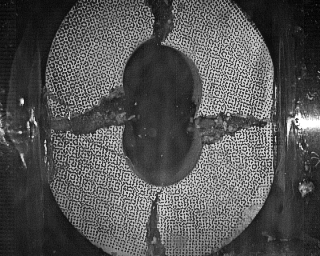


**14.62**

**15.56**

**17.34**

**16.40**

**19.98**

**16.04**

**（c）** Sample GD3-4


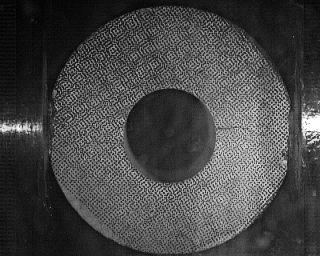

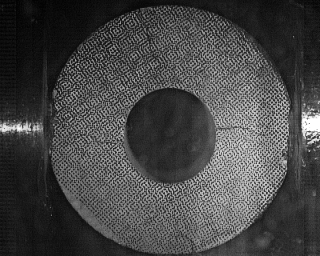

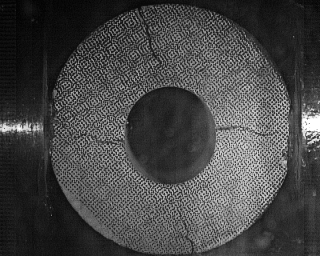

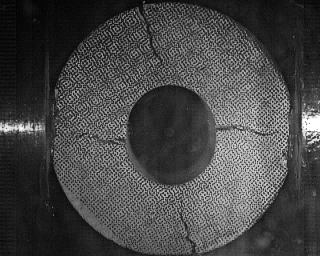

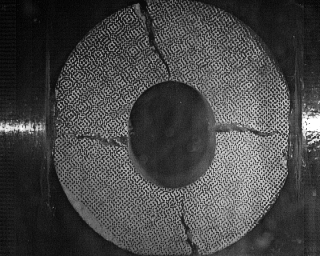

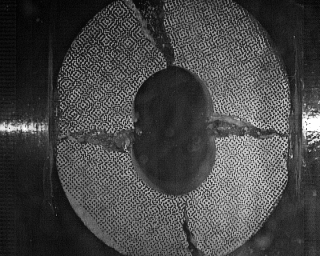


**17.02**

**17.12**

**18.56**

**18.22**

**19.98**

**17.34**

**（d）** Sample GD4-2

**Figure.7** The damage history process of ring granite under radial impact load (The number in the figure represents the moment when the camera took the photo: for example, 11.3 represents the 11.3ms when the shooting started)
