# Supplementary material for: Study on the dynamic characteristics of rock surrounding a wellbore in energy storage areas during deep geothermal energy mining
Source: PLoS One. 2020 Aug 21;15(8):e0237823. doi: 10.1371/journal.pone.0237823 (PMC7442234; doi:10.1371/journal.pone.0237823)
Supplement: S1 Data — (ZIP) [file pone.0237823.s001.zip › DATA/9+Figure 8.docx]

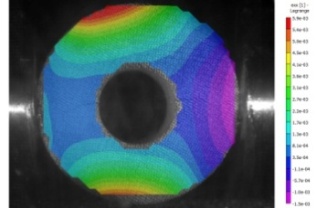

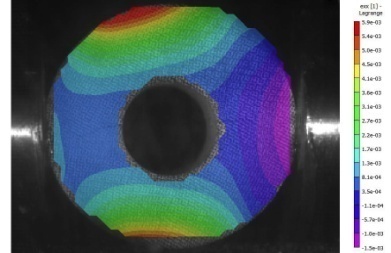

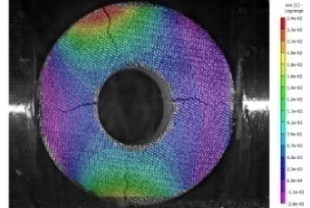

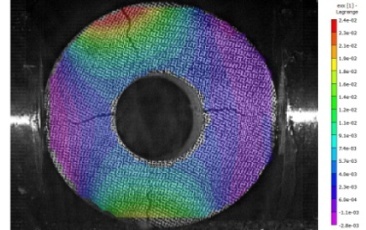

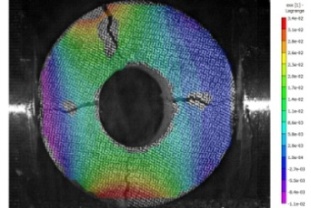

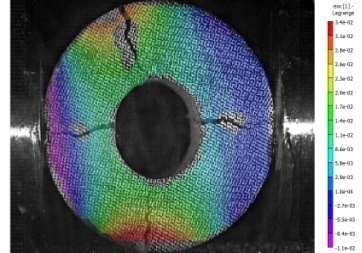

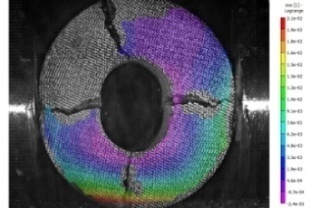

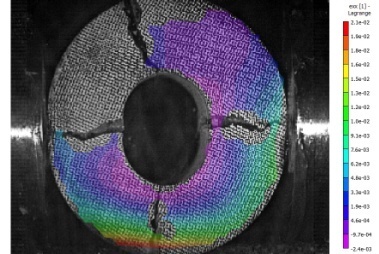

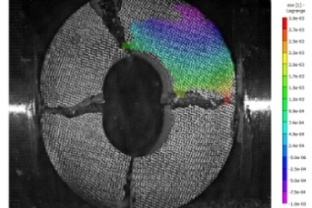

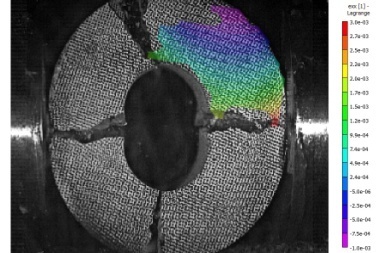

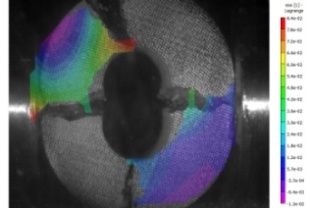

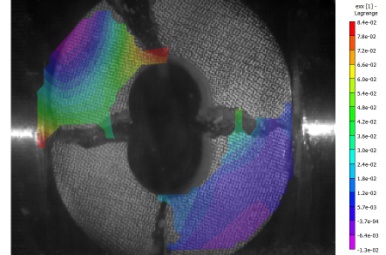


*ε_x_*:-1.5e-03~5.9e-03 *ε_x_*:-2.8e-03~2.4e-02 *ε_x_*:-1.1e-02~3.4e-02 *ε_x_*:-2.4e-03~2.1e-02 *ε_x_*:-1.0e-03~3.0e-03 *ε_x_*:-1.3e-02~8.4e-02

**（a）**The evolution of strain cloud image in x direction

(the direction of impact stress wave propagation is x direction)


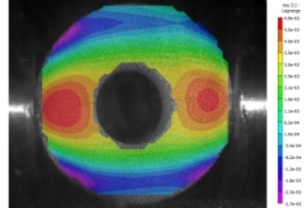

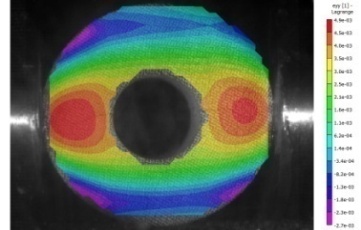

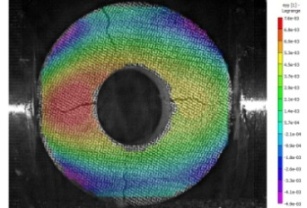

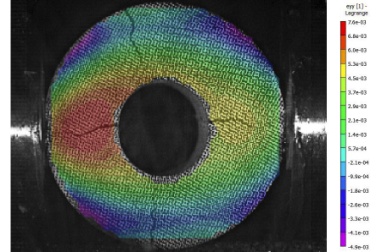

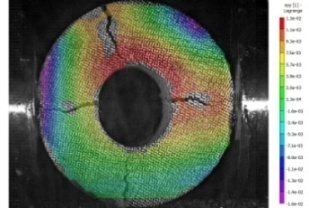

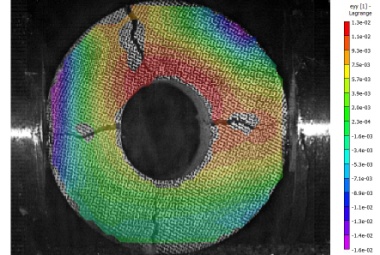

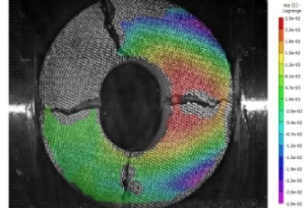

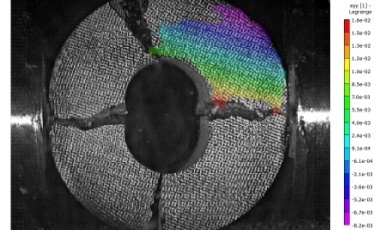

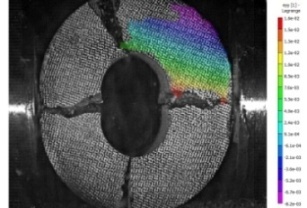

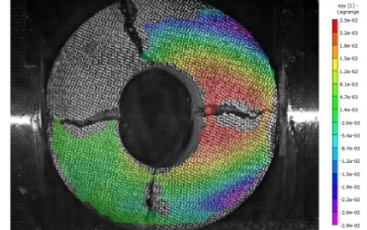

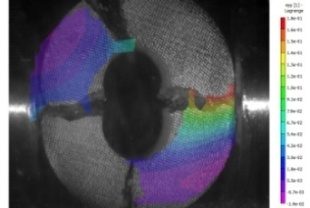

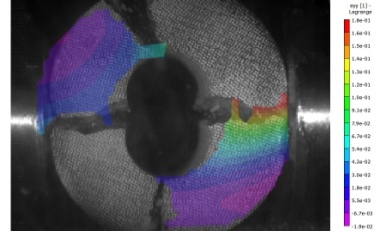


*ε_y_*:-2.7e-03~4.9e-03 *ε_y_*:-4.9e-03~7.6e-03 *ε_y_*:-1.6e-02~1.3e-02 *ε_y_*:-2.9e-02~2.5e-02 *ε_y_*:-8.2e-03~1.6e-02 *ε_y_*:-1.9e-02~1.8e-01

**（b）**The evolution of strain cloud image in y direction

(the direction of vertical impact stress wave propagation is y direction)

**Figure.8** The strain history of ring granite under radial impact load (sample GD2-2)
