# Supplementary figures and images for: Study on the dynamic characteristics of rock surrounding a wellbore in energy storage areas during deep geothermal energy mining
Source: PLoS One. 2020 Aug 21;15(8):e0237823. doi: 10.1371/journal.pone.0237823 (PMC7442234; doi:10.1371/journal.pone.0237823)

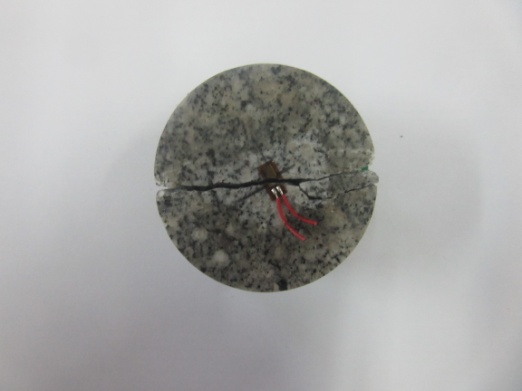

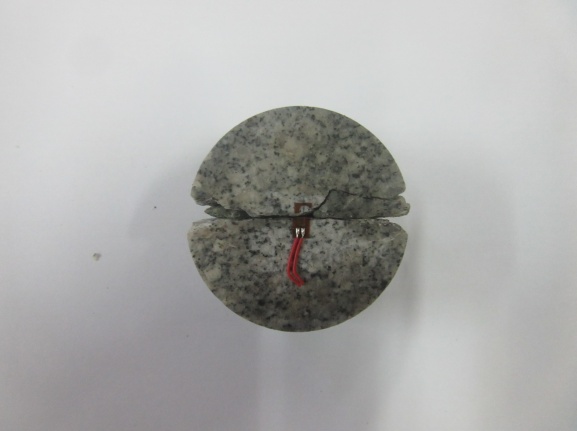

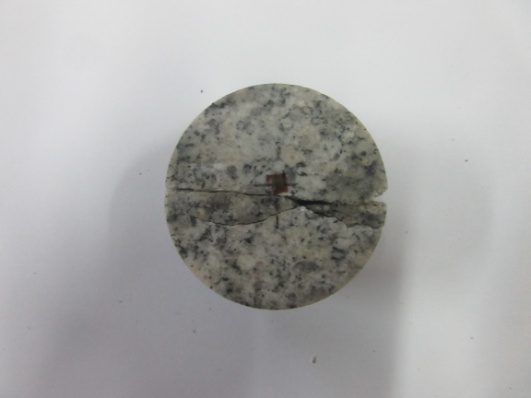


**D1**

**D2**

**D3**

**Figure.11** Failure model of Brazil disk split test

Supplement: S1 Data — (ZIP) [file pone.0237823.s001.zip › DATA/12+Figure 11.docx]

| 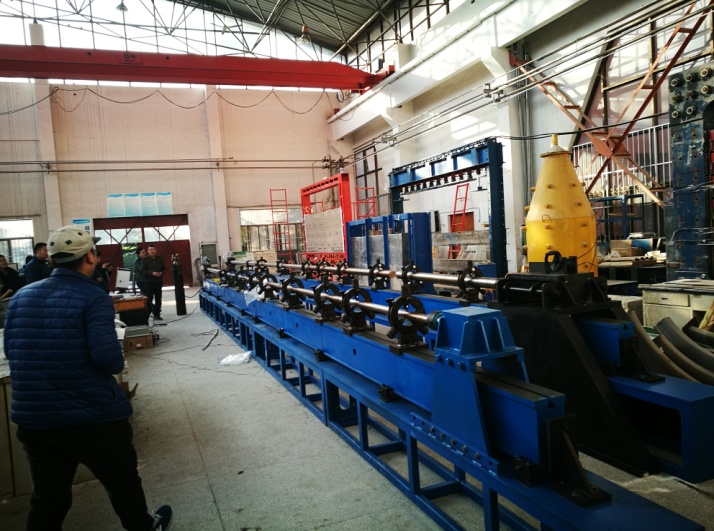  (**a**) SHPB | 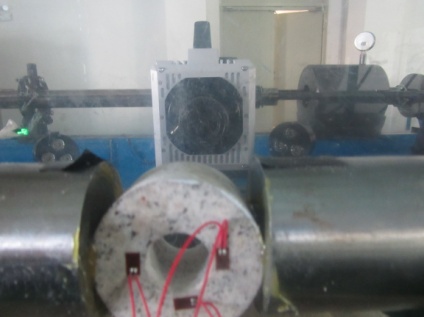  (**b**) VIC-3D |
| --- | --- |

**Figure.1** The mechanical test system with radial impact of circular granite

Supplement: S1 Data — (ZIP) [file pone.0237823.s001.zip › DATA/2+Figure 1.docx]
